# Impact of juvenile idiopathic arthritis on schooling

**DOI:** 10.1186/1471-2431-13-2

**Published:** 2013-01-07

**Authors:** Ilham Bouaddi, Samira Rostom, Dalal El Badri, Asmae Hassani, Bouchra Chkirate, Bouchra Amine, Najia Hajjaj-Hassouni

**Affiliations:** 1 Department of Rheumatology, Al Ayachi Hospital, University Hospital of Rabat-Salé, Salé, 11000, Morocco; 2 Department of Pediatrics, Children’s Hospital, University Hospital of Rabat-Salé, Rabat, 10000, Morocco

**Keywords:** Juvenile idiopathic arthritis, Children, Healthy controls, School, Absenteeism, Failure

## Abstract

**Background:**

Juvenile idiopathic arthritis (JIA) is the most common arthropathy of childhood. Different diseases affect school attendance to varying degrees. The aim of this study was to assess the impact of juvenile idiopathic arthritis (JIA) on Moroccan children’s schooling.

**Methods:**

Thirty-three children with JIA were included in this study, having been previously diagnosed according to the classification criteria of the International League of Associations for Rheumatology (ILAR). Seventy-four healthy children were recruited to serve as controls. Data was obtained for all children on their school level, educational performance, and attendance. The rate of absenteeism due to health complications was noted.

**Results:**

All healthy children were able to attend school (p<0.0001), while 33% of children with JIA were unable to attend school due to their condition. The students with JIA who were able to attend school were absent much more often than controls (63% compared to 20%), with a highly significant p value (p<0.0001). Slightly less than half of the JIA patients (48.5%) failed in their schooling. In univariate analysis, there was an association between absenteeism and tender joints (p=0.02), disease activity score (DAS28) (p=0.007), Childhood Health Assessment Questionnaire (CHAQ) (p=0.01), and erythrocyte sedimentation rate (ESR) (p=0.03). In multivariate analysis, the only association persisted between DAS28 and absenteeism.

**Conclusions:**

Our study suggested that the schooling of children with JIA was negatively impacted due to the disorder. More studies, with a larger sample of children, are needed to confirm our findings.

## Background

Juvenile idiopathic arthritis (JIA) is the most common arthropathy of childhood, with an estimated prevalence between 7 and 400 for every 100,000 children [1]. It can persist over many years and can also lead to disability and dysfunction in adulthood [2]. JIA is a heterogeneous, multifactorial autoimmune disease characterized by persistent joint inflammation, which manifests as swelling, pain, and limitation of movement [3]. The disease can also lead to physical disability and reduced quality of life [4]. Different diseases affect school attendance to varying degrees, and there are indications that chronic arthritis is particularly disruptive because of marked pain, malaise and physical restriction; therefore, the potential for school disruption is high, particularly in severely affected children [5]. Due to prolonged and/or frequent absences, children with chronic health impairments are often confronted with educational difficulties [6]. Missing school can lead to problems in keeping up with schoolwork and social relationships [7], and a prolonged absence or multiple brief absences from school may contribute significantly to negative school performance [8]. Several researchers have observed negative correlations between absenteeism (for all reasons, including illness) and academic performance [9,10]. Frequent absences from school, and thus a lack of involvement in school activities, may limit opportunities for children to establish friendships and result in increased passivity and the development of feelings of inferiority [11]. Students who are absent must compensate for potential educational disadvantages by making up assignments and utilizing home- or hospital-based educational services [12]. Increased absenteeism has been documented for a number of conditions, such as asthma, diabetes, epilepsy and hemophilia [13]. The Moroccan educational system consists of three sub-systems: subsystem school with preschool, primary, secondary and post secondary, the subsystem of higher education and the subsystem of Literacy and Non-formal Education. However, to the best of our knowledge, there is no data on Arab and/or African children with JIA concerning education. The aim of our study was to assess the impact of JIA on Moroccan children's schooling.

## Methods

Our study group included 33 children with JIA who met the classification criteria set by the International League of Associations for Rheumatology (ILAR) [14]. These children were patients of the Departments of Rheumatology and/or Pediatrics of the University Hospital of Rabat-Salé. Both departments carried out this cross-sectional study. Children were from to the region of Rabat-Sale. Any patient with a chronic disease, in addition to JIA, that would influence the child’s schooling was excluded. Children (patients and controls) less than 5 years were not included because these younger children wouldn't be expected to be in school.

The Ethics Committee of our hospital approved this study and all participants’ parents provided written consent.

A detailed questionnaire was completed for each participant by interviewing them or their parents as well as by information obtained from their medical records. Collected data included age, sex, subtype of JIA, disease duration and level of disability according to the Childhood Heath Assessment Questionnaire (CHAQ)(translated and certified in Arabic) [15]. Health status was evaluated by collecting the patient’s assessment of pain by [visual analogue scale (VAS) 0–10 cm]. The severity of JIA was assessed using the following: disease activity score (DAS28) [16,17] for polyarticular and oligoarticular JIA; Bath AS Disease Activity Index (BASDAI)(translated and certified in Arabic) for juvenile spondylarthropathy [18]; patient assessment of pain and global disease activity; physician assessment of global disease activity; erythrocyte sedimentation rate (ESR) and C-reactive protein (CRP). Medications used for JIA treatment were documented. No patient received biotherapy. This is due to the lack of social security and in Morocco only infliximab is reimbursable.

### Schooling

To assess school performance, we asked parents to indicate the child’s educational level (illiterate, primary, middle or high school) and whether or not they were currently attending school; if the child had stopped attending school, then the cause of abandonment. The number of days over the previous academic year that the child had been absent due to JIA was collected and was then measured as percentage. School failure was defined by whether the child had had to repeat a year of schooling. School level of mothers was included.

### Controls

The control group consisted of 74 children with similar age and gender to the study group. They were selected from children who had been patients in our hospital. Children with chronic diseases were excluded from the control group. They underwent physical examination and demographic characteristics were noted. Details concerning school attendance and performance were also collected. Information about the mothers of the control patients was not elicited.

### Statistical analysis

Analyses were performed using the software program SPSS for Windows (Version 13.0, SPSS Inc, Chicago, IL). Descriptive statistics were used to assess the demographic variables and characteristics of schooling. A linear regression was used to analyze the association between days absent from school and characteristics of disease (duration of disease, subtype, disease activity, and CHAQ). The Chi Square was used to compare school attendance, absenteeism and school failure of the JIA patients and healthy controls. P values less than 0.05 were considered significant.

## Results

### Characteristics of the study patients

33 patients with JIA were included, of whom 18 (54%) were male in this cross-sectional study. The median age was 11 years [5.75-14]. The median disease duration was 2 years [1–4.5]. The most common subtype was rheumatoid factor-positive polyarthritis (45.5%) followed by systematic (27.5%) and oligoarticular (22.5%). The mean DAS28 was 5.33 ±1.11. The median CHAQ was 0.5 [0–1.61]. More than half of the children (58%) were receiving corticosteroid. Disease-modifying antirheumatic drugs (DMARDs) were used by 17 of the 33 patients (methotrexate [n = 12], sulfasalazine [n = 5]). Patients’ characteristics are presented in Table 1.

**Table 1:** Socio-demographic and clinical characteristics of the patients

Female sex^1^	15(45.5)
Age (years)^2^	11[5.75-14]
Subtype of AIJ^1^	
Systemic-onset arthritis	8(24.2)
Oligoarthritis	4(12.1)
Rheumatoid factor- positive polyarthritis	15(45.5)
Rheumatoid factor- negative polyarthritis	1(2.5)
Enthesitis-related arthritis	5(15.2)
DAS28^3^	5.33 ±1.11
CHAQ^2^	0.5 [0-1.61]
BASDAI^2^	0.7 [0-2.75]
ACPA Positive^1^	5(15.2)
Rheumatoid factor positive (RF)^1^	4(12.1)
Antinuclear antibodies positive^1^	25(76)
ESR (mm/h)^2^	35[25-50.5]
CRP (mg/l)^2^	20[10.5 -40]
Medications used :	
NSAID^1^	26(79)
Corticosteroid^1^	19(58)
Methotrexate^1^	12(36.4)
sulfasalazine^1^	5(15.2)
**case-control:**	
Female sex^1^	33(45)
Age (years)^2^	10[6-14]

### Characteristics of patients’ schooling

**Figure 1:**
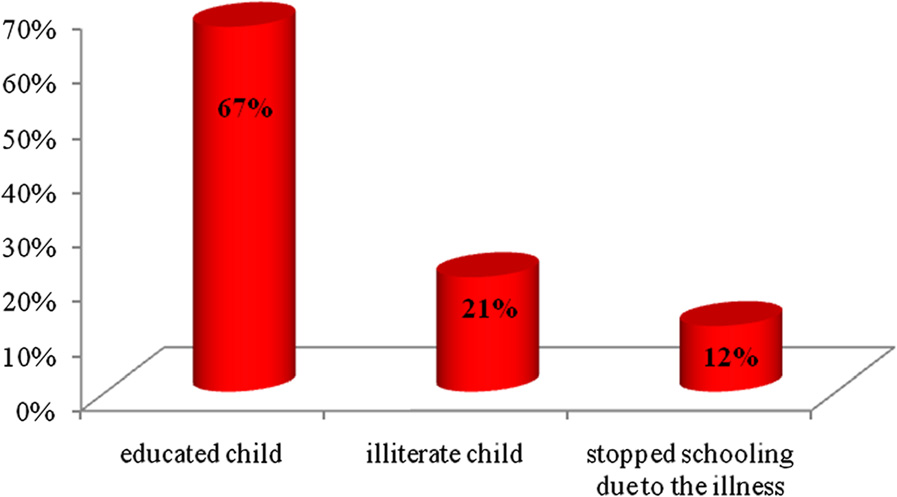
Schooling profile of patients.

Sixty-seven percent of JIA patients were able to attend school while 12% had to stop their schooling and 21% were illiterate because of their illness (Figure 1). A year of schooling was repeated by 48.5% of patients. The median absenteeism rate was 2% [0–6].

### Variables associated with absenteeism and school failure

In univariate analysis, there was an association between absenteeism and tender joints (p = 0.02), DAS28 (p = 0.007), ESR (p = 0.03) and CHAQ (p = 0.01). In multivariate analysis, a positive association existed between DAS28 and absenteeism (Table 2). School absence was not significantly associated with age, JIA subtype, duration of illness, treatment and/or swollen joints (Table 2). 51.5% of mothers were illiterate. 81% of children, who had failed in their schooling, their moms were illiterate. The failure of children in school was significantly linked to illiterate mothers (p = 0.001). There were no association between school failure and sex (p = 0.6).

**Table 2:** Variables associated with absenteeism

	***Univariate analysis***			***Multivariate analysis***		
	***B***	***IC***_***95%***_	***P***	***B***	***IC***_***95%***_	***P***
Age (year)	0.24	[-0.36-1.48]	0.22			
Subtype of JIA	-0.03	[-4.65-4.01]	0.8			
Duration of disease (year)	0.28	[-0.45-2.88]	0.14			
Tender joints	0.44	[0.13-1.57]	**0.02**	-0.75	[-1.89-0.48]	0.2
Swelling joints	0.29	[-0.37-2.48]	0.14			
ESR (mm/h)	0.41	[0.01-0.38]	**0.03**	-0.70	[-0.48-0.12]	0.2
CRP (mg/l)	0.27	[-0.06-0.35]	0.16			
DAS28	0.70	[0.93-4.63]	**0.007**	1.91	[0.61-14.47]	**0.03**
BASDAI	-0.18	[-0.28-0.20]	0.6			
CHAQ	0.44	[0.80-8.14]	**0.01**	0.29	[-1.41-4.39]	0.2

*DAS*, disease activity score; *CHAQ*, Childhood Health Assessment Questionnaire; *ESR*, Erythrocyte sedimentation rate; *CRP*, C-reactive protein; *BASDAI*, Bath AS Disease Activity Index.

### Characteristics of controls’ schooling

The median age was 10 years [6-14] and all children attended school. No child in this population had to repeat a year of schooling. The median absenteeism rate was 0.68% [0.68-1.02].

### Comparison between patients and healthy controls

Only 67% children having JIA attended school while all healthy children did (p < 0.0001). Those children with JIA who did attend school were absent much more often than the control (63% compared to 20%) (p < 0.0001), and 48.5% of patients failed in their schooling (p < 0.001). Twelve percent of patients stopped their education because of the disease versus 0% for controls (p < 0.0001) (Table 3).

**Table 3:** Comparison between patients and healthy controls

	**Healthy controls**	**JIA**	**p**
Schooling	100%	67%	**<0.0001**
Absenteeism	20%	63%	**<0.0001**
Schooling failure	0%	48.5%	**<0.0001**
The school stop	0%	12%	**<0.0001**

## Discussion

The main objective of this study was to determine the impact of JIA on Moroccan children's schooling. Our study showed that JIA negatively affects schooling. All healthy controls were able to attend school, compared to 33% of patients with JIA who were unable to do so. For a child with a chronic disease, such as JIA, the school environment may be fraught with problems [19-22]. Two studies noted that in academic achievement, chronically ill school-age children perform below their peers [21,22].

Our study showed that the percentage of patients with JIA who left school was high compared to the healthy controls, and in addition they missed more school days per year. Sturge et al. found that children with JIA had an overall mean school attendance rate of 92% (equivalent to 15 days absent per year), which was comparable to rates described in another study of children with a chronic physical illness such as asthma [23]. Barbara et al. studied children with a chronic illness, and found that the mean number of days absent was 16.9 and the mean percentage of days absent was 9.4% [24]. There is evidence suggesting that absenteeism plays a large role in determining students’ achievement on both standardized tests and classroom performance, which our work further supports [25]. A study of boys with hemophilia/HIV disease conducted by Indiana State University assessed the effects of absenteeism on cognitive skills index and various achievement indicators and found that Hemophilia may be a risk factor for academic underachievement [26]. In our study, almost half of the children with JIA (48.5%) had failed in their schooling, while this percentage was zero for healthy controls (p < 0.0001). Children and adolescents with chronic illness experience more academic difficulty than their healthy peers [27]. In a different study, 45% of students with chronic illness reported falling behind in their schoolwork, which lead them to dislike school [28]. Our study showed that children’s failure at school was significantly associated with illiterate mothers (p = 0.001). This can be explained by the fact that parents who did not complete school may place less value on education and school attendance. Only the parents’ education emerged as a possible protective factor [29]. The parents’ education may act as a proxy indicator of the value families place on education, the expectations they have for academic achievement, and/or a genetic predisposition for academic achievement [29].

Additionally, chronic absenteeism would affect the child’s ability to cope with their illness because it would interfere with the child learning the necessary skills to achieve a productive life [24]. We established that disease activity and CHAQ were the two significant factors that predicted the number of days missed from school in univariate analysis. In the study by Sturge et al., a small group of four children (all suffering from the more severe poly illness type) had missed a great deal of schooling (between a quarter and a half of the time) and nine (or about one in 10 of the whole group) had missed >20% of the time (39 days) [7]. As in previous studies with ill children [5,23,30-32], Sturge et al. found that attendance was linked to the severity of illness, in as much as this can be ascertained through type of illness (pauci- and polyarthritis), and was more compromised in the poly- group. We, however, didn't find an association with absenteeism and each specific subtype of JIA. Our study showed that there is a positive association with absenteeism and CHAQ. Miller et al. completed a retrospective study where school age children with JIA (with and without manifest synovitis) were compared with healthy controls. He reported the loss of physical functioning (CHAQ) at between 10% and 65% [33]. If the child’s hands were affected, simple things such holding a pencil or writing for extended periods of time would be painful, making it more difficult to complete class work in a timely manner [33].

Despite the methodological limitations of this study (cross-sectional, single-center), this study has two strengths absence of information about the mothers of the control patients. First, it could support the few studies concerning the schooling of children with JIA. Second, this study is the first one performed in an Arab and/or African country.

## Conclusions

Our study suggests that children's schooling is affected by JIA. Disease activity and functional impairment seem to influence the school level of children, hence the importance of proper management of these patients to increase their academic performance. Additional research, with a larger sample of children, is needed to confirm our findings.

## Competing interest

The authors declare that they have no conflict of interest.

## Authors’ contributions

N. Hajjaj-Hassouni, B. Amine, S. Rostom: correction section of the article. D. El Badri, A. Hassani, B. Chkirate: collection of patients and fill the questionnaire All authors read and approved the final manuscript.

## Pre-publication history

The pre-publication history for this paper can be accessed here:

http://www.biomedcentral.com/1471-2431/13/2/prepub
